# A multi-national observational study on the concordance between the translational triage tool and routine prehospital triage

**DOI:** 10.1038/s41598-026-52015-7

**Published:** 2026-05-07

**Authors:** Phatthranit Phattharapornjaroen, Amir Khorram-Manesh, Zakaria Mani, Katarzyna Naylor, Adel Ahmed Darraj, Eric Carlström, Yuwares Sittichanbuncha, Krzysztof Goniewicz

**Affiliations:** 1 Paramedic School, Faculty of Health Science Technology, Chulabhorn Royal Academy, Bangkok, Thailand; 2 HRH Princess Chulabhorn Disaster and Emergency Medicine Center, Chulabhorn Royal Academy, Bangkok, Thailand; 3 Department of Surgery, Sahlgrenska Academy, Institute of Clinical Sciences, University of Gothenburg, Gothenburg, Sweden; 4 Center for Disaster Medicine, University of Gothenburg, Gothenburg, Sweden; 5 Gothenburg Emergency Medicine Research Group (GEMREG), Sahlgrenska University Hospital, Gothenburg, Sweden; 6 Nursing Department, Jazan University, Jazan, Saudi Arabia; 7 Independent Unit of Emergency Medical Services and Specialist Emergency, Medical University of Lublin, Chodzki 7, Lublin, 20-093 Poland; 8 Emergency Department, King Fahad Central Hospital - Jazan, Jazan Cluster, Jazan, Saudi Arabia; 9 Department of Security, Polish Air Force University, Dęblin, Poland

**Keywords:** Mass casualties, Trauma, Prehospital, Triage, Translational triage tool, Health care, Medical research

## Abstract

Effective and standardized prehospital triage is crucial in mass casualty incidents (MCIs) to optimize patient outcomes. Existing triage systems often vary by region, creating challenges for multinational disaster response. A “translational” tool, intended to support interoperability and potentially bridge routine and MCI contexts, has been proposed. As a preliminary step, this study evaluates the concordance, defined here as the post-hoc categorical agreement) of the translational triage tool (TTT) with established, routine triage systems across diverse international settings. This multinational observational study included 301 patients from Poland, Saudi Arabia, and Thailand. Agreement between the routine triage outcome and TTT categorization applied by the observer was assessed within each country using Cohen’s kappa (linear and quadratic weights). For sites using five-level routine systems (CTAS / ESI), categories were pre-specified and collapsed into a three-level framework for comparison (CTAS 1 = Red; CTAS 2 = Yellow; CTAS 3–5 = Green; ESI 1 = Red; ESI 2 = Yellow; ESI 3–5 = Green). Cross-tabulations were used to describe patterns of discordance (over- and under-triage relative to the local routine triage system). No inferential comparisons between the tools were performed beyond agreement estimates because the data were categorical/ordinal and pooled analyses were not defensible given heterogeneity across sites. Agreement levels varied across countries: complete categorical concordance in Poland (kappa = 1.000), substantial in Saudi Arabia (kappa = 0.810), and good in Thailand (kappa = 0.701). Discordant cases in Saudi Arabia and Thailand suggested that the physiologically focused TTT—more often assigned lower urgency to patients with stable physiology but high-risk presentations or medical histories, while sometimes assigning higher urgency when respiratory signs appeared abnormal. The TTT shows strong alignment with the physiologically oriented START approach but diverges from complaint- and risk-modifier-driven systems such as CTAS and ESI, particularly when assigning of lower urgency to some high-risk presentations without overt physiological compromise. This study assesses post-hoc concordance rather than clinical accuracy or patient outcomes; therefore, the findings should not be interpreted as validation of TTT for routine clinical use. The pattern of agreement with START and discordance with CTAS/ESI systems is consistent with a physiologically driven disaster triage approach, and future studies should evaluate the TTT specifically in surge / MCI contexts, including operational feasibility and outcome-based safety.

**Supplementary Information:**

The online version contains supplementary material available at 10.1038/s41598-026-52015-7.

## Introduction

The landscape of emergency medical services (EMS) and disaster response is inherently dynamic, characterized by the unpredictable nature of medical emergencies and, more acutely, by the sudden overwhelming demands of mass casualty incidents (MCIs). In these critical scenarios, the ability to rapidly assess, prioritize, and allocate scarce resources is paramount to mitigate loss of lives and optimize patient outcomes^1,2^. At the heart of this complex process lies triage, a systematic approach to categorizing patients based on the severity of their injuries or illnesses and the urgency of their need for medical attention^1–3^. While routine, daily triage in emergency departments efficiently manages patient flow, the exigencies of MCIs, ranging from natural hazard- induced disasters and large-scale accidents to acts of terrorism, and from prehospital to hospital settings, require adaptable and highly effective triage strategies^4,5^. This distinction highlights the critical need to separate transitional triage—the operational shift in triage approach during surge or MCI conditions—from translational triage tools, which are proposed instruments intended to support interoperability across systems and potentially bridge routine and disaster contexts^4,6,7^.

The concept of translational triage tools broadly refers to tools intended to bridge the gap between routine medical operations and the escalated demands of a surge event or MCI. It acknowledges that the principles of triage must evolve as the patient load exceeds the available resources, shifting from an individual-focused approach to a population-based strategy that prioritizes the greatest good for the greatest number^6,7^. Such transitions often involve “reverse triage,” where stable inpatients might be discharged to free up beds for incoming casualties, or the rapid deployment of simplified assessment tools to quickly sort a large influx of victims^8^. The ‘translational’ concept, as we define it, posits that a single, scalable tool, used daily, would build responder familiarity and prevent the cognitive-load failure seen when a separate ‘disaster-only’ tool is suddenly activated. However, the viability of such a translational tool is unknown. A major, unanswered question is whether a tool *simple* enough for a disaster (such as a physiologically oriented algorithm) is *robust* enough for daily use. A tool that cannot safely manage routine, diagnostically complex emergencies (e.g., diabetes ketoacidocis (DKA), stroke, sepsis) that may present with stable vital signs would be a failure in daily practice. Before any such tool can be tested for implementation, we must first understand how it *maps* onto the existing, validated routine tools.

The objective remains consistent: to ensure that limited resources are directed towards those who will benefit most, thereby maximizing survival and minimizing morbidity across the affected population. The efficacy of these translational strategies is not limited to large-scale disasters; it extends to daily challenges in emergency departments facing overcrowding, where efficient patient flow and resource optimization are continuous imperatives^9^. The overarching goal is to standardize the chaotic situation, enabling effective communication and decision-making under duress, thus enhancing patient safety and reducing the overall burden on the healthcare system^10^.

Despite the universal acceptance of triage as essential in emergency medicine, a significant challenge persists in the prehospital setting during MCIs: the heterogeneity of existing triage systems. Numerous primary triage tools, such as START (Simple Triage And Rapid Treatment), Sieve, SALT (Sort, Assess, Life-saving Interventions, Treatment/Transport), CTAS (Canadian Triage and Acuity Scale), ESI (Emergency Severity Index), and others, are employed globally^3,11^. While each possesses merits, their diversity can lead to inconsistencies in patient categorization, impede interoperability between different responding agencies (especially in multinational or multi-agency disaster responses), and complicate data collection and analysis. This fragmentation underscores the urgent need for a more standardized, universal approach that can be readily understood and applied by a diverse range of responders, from paramedics and firefighters to military personnel and civilian volunteers^3,12,13^. This is a challenge that the concept of the “Translational Triage Tool” (TTT) aims to address. The TTT research program is not merely about introducing another new triage system; rather, it aims to develop a candidate harmonization framework that can support more consistent categorization across settings during MCI events.

**Fig. 1:**
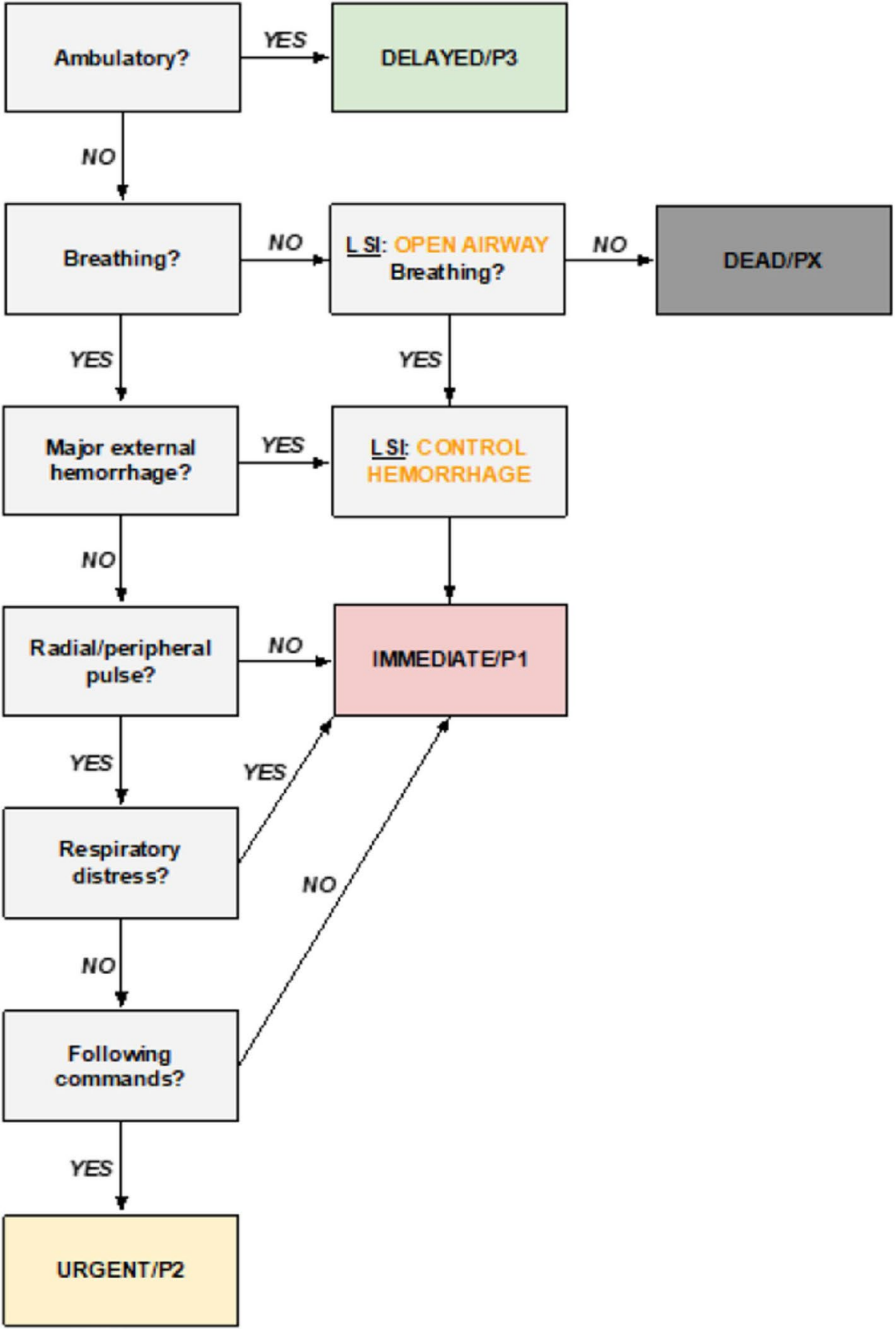
Shows the TTT algorithm.

The TTT has been described previously in a staged research program including conceptual development, expert consensus refinement, and simulation-based comparisons with existing disaster triage approaches (Fig. 1)^4,6,7^. Those studies primarily assessed categorical assignment patterns and agreement under simulated conditions rather than patient-outcome validation and therefore do not establish clinical accuracy. The present study extends this work by examining post-hoc concordance between TTT categorization and routine prehospital triage systems applied during real patient encounters in three countries^4,6,7^.

Accordingly, the aim of this study was to assess post-hoc categorical agreement (concordance) between the TTT and existing routine triage systems in diverse prehospital settings, and to describe the direction and clinical context of discordant assignments.

## Methods

### Study design and setting

**Fig. 2:**
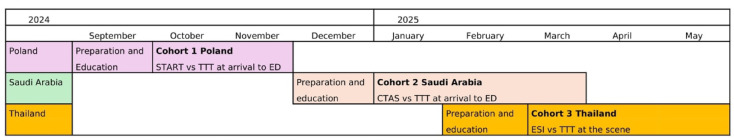
Shows the study period in different countries.

This prospective, observational, multi-center concordance study aims to evaluate the post-hoc categorical agreement of the TTT with routine prehospital triage practices across diverse international settings. The study was carried out over an eight-month period (October 2024 to May 2025, in three distinct geographic and healthcare contexts (Fig. 2). Poland (representing Europe, START triage), Thailand (representing East Asia, ESI triage), and Saudi Arabia (representing the Middle East, CTAS triage). The selection of these countries ensures a representation of various prehospital emergency medical service (EMS) systems, patient demographics, and routine triage protocols, providing a robust platform for comparative analysis.

The protocol of this study was registered in October 2024 at Figshare with the following Doi number: 10.6084/m9.figshare.26840494.v1. The raw material used in this study for presentation and statistical analysis are included as supplementary files.

### Research teams and working process

In each participating country, a dedicated research team (2–3 members) with at least one expert in triage, led by a designated research leader was established. Before data collection began, all research personnel involved in applying the TTT underwent standardized training (workshop training covering algorithm application and scenario-based drills) to ensure consistent application of the tool’s algorithm and data recording procedures. This training covered the principles of the TTT, its step-by-step application, and detailed instructions for data capture, including the use of a standardized data collection form (Fig. 2).

One designated member of each research team accompanied EMS crews on all emergency calls within the study’s designated operational areas, irrespective of the initial presenting complaint or suspected diagnosis. This continuous presence ensures comprehensive data capture across a wide spectrum of emergency situations. At the scene of each emergency call, the routine prehospital triage was performed by the local EMS staff according to their established national or regional protocols.

### Data collection, blinding and inter-rater reliability

#### Data collection

After the primary EMS crew had completed their routine triage and determined the patient’s care path, the designated research team member (who was not involved in patient care) applied the TTT to the patient at scene, during transport or upon arrival at the emergency department (ED), without influencing or interfering with the routine triage process performed by the EMS personnel. This was an observational application and the TTT outcome was not used for any clinical decision-making. Both results were recorded. In addition to triage categories, relevant demographic data (e.g., age, sex) for each encountered patient were collected.

#### Blinding

Observers applying the TTT were not blinded to the routine triage category at the time of TTT assignment. This may have introduced contextual bias, and potentially inflated agreement estimates and is addressed as a limitation.

#### Inter-rater reliability

One trained observer per site applied the TTT. Formal inter-rater reliability testing between multiple TTT raters was not performed and is a priority for future work.

### Data management and statistical analysis

All collected data from the three participating countries were compiled into a secure, centralized database. Data cleaning and validation procedures were performed to ensure accuracy and completeness.

#### Handling of missing data

Cases missing, either the routine triage category, or the TTT category were excluded from concordance analyses (complete-case analysis), because category-pairing is required for kappa estimation. 

#### Harmonization of routine triage scales to a 3-level comparison framework

 Routine triage systems differed across sites. To enable comparison with the three-level TTT output (Red/Yellow/Green; with Black where applicable), we pre-specified the following mapping:

- CTAS 1 = Red; CTAS 2 = Yellow; CTAS 3–5 = Green.
- ESI 1 = Red; ESI 2 = Yellow; ESI 3–5 = Green.

We acknowledge that collapsing five categories into three introduces assumptions and loss of information; alternative thresholds could change observed agreement. Therefore, agreement estimates are interpreted as post-hoc concordance under the stated mapping, not as validation of clinical accuracy.

#### Agreement statistic

The primary objective of the statistical analysis was to assess the agreement between routine prehospital triage practices in each country and the TTT. For this, Cohen’s Kappa (κ) statistic was employed (both linear and quadratic). Kappa coefficients were calculated for each country independently to evaluate the level of agreement (e.g., poor, slight, fair, moderate, substantial, almost perfect) between the two triage methods. Where feasible, 95% confidence intervals (CIs) for kappa are reported.

Given the significant heterogeneity in routine triage tools (START, CTAS, ESI), population, and healthcare systems, an aggregate or pooled Kappa coefficient across all countries was deemed methodologically inappropriate (primary inference)^14,15^. Linear kappa treats all disagreements equally, while quadratic kappa gives more weight to larger disagreements. For triage a good quadratic kappa is particularly important as it reflects the clinical severity of misclassifications^15^. However, we report an “overall” kappa computed on the pooled dataset after harmonization as a descriptive summary only. This pooled metric should be interpreted cautiously because it aggregates heterogeneous systems and case-mix, and it does not replace site-specific analyses. Cross-tabulations were used to describe concordant and discordant cases, including the direction of disagreement (over-/under-triage relative to the local routine system).

Furthermore, demographic characteristics of the patient populations encountered in each country were summarized using descriptive statistics (e.g., means, and standard deviations for continuous variables like age, and frequencies and percentages for categorical variables like sex).

### Ethical considerations

Ethical approval for this study was obtained from the relevant and involved institutional review boards or ethical committees (Saudi Arabia ‘s ethical approval number No. 24113, and Thailand’s ethical approval number; EC 139/2567) (Appendices 1 and 2).

In Poland, according to applicable national regulations, this type of study does not require approval from an Institutional Review Board or Bioethics Committee. Under Polish law, ethical approval is mandatory only for medical experiments or studies involving medical interventions. The present study did not meet the legal definition of a medical experiment and therefore was exempt from ethics approval requirements. Specifically, under the Act of 5 December 1996 on the professions of doctor and dentist (Journal of Laws 1997 No. 28, item 152, Article 29a), as well as the Pharmaceutical Law Act of 6 September 2001 (Journal of Laws 2020, item 944, Article 37 L), ethical committee approval is not required for non-interventional, anonymized observational studies. Consequently, approval from an Institutional Review Board (including the Medical University of Lublin) was not required for the Polish part of the study.

Due to the observational nature of the study and the focus on anonymized triage categorization rather than direct patient intervention, individual patient consent was waived, or a deferred consent process was implemented in accordance with local ethical guidelines, ensuring that patient care remains the primary focus and no personal identifying information was collected. All data were handled with strict confidentiality and in compliance with data protection regulations (e.g., GDPR in Europe, and equivalent regulations in Thailand and Saudi Arabia).

## Results

### Demography

A sum of 301 patients was included in this study. The distribution among involved countries were Poland (*n* = 100), Saudi Arabia (*n* = 101), and Thailand (*n* = 100). There was a variation in the age of the included patients with Saudi Arabia having the youngest group of patients. Both Poland and Thailand had a mean age of over 60 years, while Saudi Arabia patients had a mean age of 29.26 years (Table 1). This heterogeneity reflects different case-mix and limits cross-country comparability of triage patterns.

**Table 1:** The age distribution of included patients from the three research centers.

Country	*n*	Mean age	Median	SD	IQR	Min	Max
Poland	100	63.69	68.50	19.62	26.00	18.00	95.00
Saudi Arabia	101	29.26	26.00	19.52	25.00	1.00	78.00
Thailand	100	60.46	60.50	21.15	27.00	0.00	100.00
Total	301	51.06	54.00	25.38	41.00	0.00	100.00

All countries demonstrated more males in their data, even though Thailand showed a more balanced distribution with 45% female compared to 39% in Poland and 33% in Saudi Arabia (Table 2).

**Table 2:** Shows the distribution among the researched countries with Thailand having a higher number of females.

Country	Gender cleaned	Count	Proportion (%)
Poland	Male	61	61.0
Poland	Female	39	39.0
Saudi Arabia	Male	68	67.3
Saudi Arabia	Female	33	32.7
Thailand	Male	56	55.4
Thailand	Female	45	44.6

Table 3 shows concordant and discordant triage assignments. In Poland, the sample included only “Red” and “Yellow” categories; therefore, kappa and “complete concordance” should be interpreted within this restricted category range. In Saudi Arabia and Thailand, discordances reflected both over-triage and under-triage relative to local routine triage.

**Table 3:** The comparative results of 301 cases between routine triage and the TTT.

Country	Routine Triage Outcome	TTT Outcome	*n*	Deviated cases
Poland	Red	Red	35	
Poland	Yellow	Yellow	65	
Saudi Arabia	Green	Green	36	
Saudi Arabia	**Green**	**Yellow**	**5**	Fever x 2, Headache, Crohn disease, Liver Cirrhosis
Saudi Arabia	Red	Red	13	
Saudi Arabia	**Red**	**Yellow**	**7**	Testis torsion, Diabetic Ketoacidosis (DKA), Upper Gastrointestinal Bleeding (UGIB), Chest infection, Fever, Suspect TB, missed abortion
Saudi Arabia	Yellow	Yellow	40	
Thailand	Black	Black	1	
Thailand	Red	Red	78	
Thailand	**Red**	**Yellow**	**5**	Trauma, Syncope, Depression, Chest pain, Limb weakness
Thailand	**Red**	**Green**	**1**	mental deterioration
Thailand	Red	Black	1	
Thailand	Yellow	Yellow	9	
Thailand	**Yellow**	**Green**	**3**	Dialysis patient, Unconscious still breathing, Limb weakness
Thailand	Green	Green	3	

### Qualitative context for discordance (not outcome validation) (Table 3)

There was a complete agreement between the TTT results and routine triage in Poland (START). Meanwhile, the patients included in Saudi Arabian data were categorized as red, yellow and green. In 5% of cases, patients labeled as green by routine triage were categorized as yellow by the TTT (i.e., overtriage by the TTT), while 7% of patients labeled as red in routine triage (CTAS) were cataegorized as yellow by the TTT (undertriage). In Thailand, the inconsistency between the TTT and routine triage (ESI) increased by 7% undertriage in TTT labeled patients. Five patients were classified as red by routine triage but yellow by the TTT, one patient was classified red by routine triage but green according to the TTT (6%). One patient was labeled red by routine triage but black by the TTT. These cases were further investigated separately by looking at the diagnosis.

- Saudi Arabia: The TTT overtriaged five cases compare with the routine triage. Two cases were 3- and 12-years old individual who ask for help due to fever. Another patient had a Crohn disease, one suffered from liver cirrhosis, and one had headache. In all cases the main issue seemed to be abnormal breathing or hyperventilation, which led to an overtriage by the TTT. In seven cases, the TTT undertriaged patients from Red to Yellow. One had testis torsion, another had Diabetic Ketoacidosis (DKA), a third patient had Upper Gastrointestinal Bleeding (UGIB), and the remaining three had fever, suspect Tuberculosis (TB), and missed abortion respectively. All these cases appeared to be undertriaged by the TTT breathing difficulties were abscent.
- Thailand: The TTT undertriaged nine cases, compared with the routine triage. Five cases were undertriaged from Red to Yellow. These include one trauma case, one syncope, one with depression, one with chest pain and one with limb weakness as part of cerebrovascular disease. One case with mental deterioration, labeled as Red by routine triage, was cataegorized as Green by the TTT. Other cases (*n* = 3) with limb weakness, medical deterioration under dialysis treatment, and one unconscious patient with no breathing issues were labeled Yellow by routine triage and Green by the TTT (Table 3).

### Presenting medical diagnosis

Investigating the diagnostic outcomes, the results show a variety of diagnosis across diverse disciplines. In total, 23 cases of cardiac arrest, 61 cases of trauma, 57 cases of cardiopulmonary diseases, 83 neurological cases, 36 cases of abdominal conditions were among the collected data. Forty-one cases were labeled as others, consisting of fever, intoxication, etc. (Table 4).

**Table 4:** Diagnosis of included patients from all three countries.

Preliminary diagnosis/cause of call (*n* = 301)		
Cardiac arrest		**23**
Trauma/accident		**63**
Accident (12), Head injury (7), Fracture (6), Trauma (32), Traumatic cardiac arrest (1), Traumatic Pneumothorax (1), Wound injuries (2), Hemothorax (1), Shoulder dislocation (1).		
Cardiovascular and Pulmonary related		**55**
Dyspnea/Shortness of breath (19), Chest pain (6), ACS (3), Arrythmia (3), Cardiac failure and Pulmonary oedema (4), Aorta stenosis (1), ARDS (1), Respiratory failure acute or chronic (2), Cold and cough (5), Pneumonia (2), Chest infection (3), Suspect TB (1), Hypertension (3), Limb ischemia (2).		
Neurological related		**83**
Mental deterioration/depression (19), Cerebral hemorrhages (8), Syncope/unconsciousness (13), Cerebral infarction/stroke/TIA (26), Seizure (6), Headache (8), Meningitis (1), MS (1), Radial nerve damage (1).		
Abdominal related		**42**
Acute abdomen (4), Aorta aneurysm/rupture/dissection (5), Appendicitis (1), Ileus (1), Testis torsion (1). Acute peptic ulcer with hemorrhage/UGIB/gastritis/acute pancreatitis (8), Vomiting (3), Liver cirrhosis/Ascites (4), Bowel ischemia (1), Crohn’s disease (1), Chronic diarrhea (1), CKD/ESRD (3), Urogenital (2), Gynecological (7).		
Others		35
Fever (15), Infectious diseases (6), Inflammatory diseases (1), Allergic reaction (2), Anemia (1), DKA (1), Electrolyte disturbances (6), Electrocution (1), Suicide (1), Acute intoxication (1).		

### Statistical analysis

#### Cohen’s kappa

As described in the method section, the agreement between the TTT and routine triage was assessed for each country by using Cohen’s kappa (Linear as well as Quadratic). An overall pooled kappa is reported as descriptive only and should be interpreted cautiously. In the context of triage—where misclassifying a patient from *Red* to *Yellow* is less severe than from *Red* to *Green*—quadratic weighting is more appropriate than unweighted or linear kappa. It provides a clinically meaningful interpretation of misclassification risk. Our results showed a high overall quadratic kappa of 0.918, indicating almost perfect agreement between the two systems. When stratified by country, the agreement remained robust: Poland demonstrated perfect agreement (κ = 1.00), followed by Saudi Arabia (κ = 0.88), and Thailand (κ = 0.78) (Table 5). These findings support the consistency of the TTT across diverse EMS environments and reinforce its potential as a standardized triage tool for multinational or cross-border emergency response operations.

**Table 5:** Shows the result of the statistics conducted for this study.

Country	Linear Cohen’s kappa	Quadratic Cohen’s kappa	95% CI (Linear)	95% CI (Quadratic)
Poland	1.000	1.000	1.000	1.000
Saudi Arabia	0.841	0.881	0.748–0.919	0.806–0.938
Thailand	0.701	0.783	0.572–0.876	0.602–0.918
Overall	0.898	0.918	0.853–0.938	0.875–0.950

Poland had only Red/Yellow categories in this sample, which can inflate kappa due to restricted variability.

## Discussion

This prospective, multi-center observational study provides preliminary descriptive data on the post-hoc concordance of the TTT compared to established routine triage systems in Poland (START), Saudi Arabia (CTAS), and Thailand (ESI). The primary finding is that agreement varied by routine triage philosophy. Importantly, this study assesses concordance, not diagnostic accuracy, patient outcomes, or operational performance.

### Key interpretation: triage philosophy differences

The TTT is primarily a physiologically driven tool, heavily weighting observable signs like breathing, and consciousness. This is broadly consistent with disaster triage logic and the simple, physiologically based START triage^4,16^. In contrast, CTAS and ESI incorporate chief-complaint pathways, risk modifiers, and/or anticipated resource needs, which may assign higher urgency to high-risk presentations even when physiology is temporarily stable^17,18^. This could be an issue if the TTT was proposed as a routine triage system. However, using the TTT in resource-limited situations, such as disasters and public health emergencies, may yield to other considerations. The discordances observed in Saudi Arabia and Thailand—especially assignments of lower urgency to time-sensitive or high-risk presentations without overt physiological compromise—are consistent with these philosophical differences. The following paragraphs analysis the outcomes of this study in routine care and disaster care settings.

### Detailed analysis by country and key finding (routine care)

#### Poland: perfect agreement with START triage (κ = 1.00)

The observed complete concordance suggests that the TTT and the START system share an almost identical underlying logic. START is designed for MCIs and is based on a simple, stepwise assessment of (1) Ability to walk (Minor/Green), (2) Respirations (Present/Absent, Rate), (3) Perfusion (Capillary Refill/Pulse), and (4) Mental Status (Follows Commands)^4,16^.

The fact that TTT aligns exactly indicates it likely follows a similar, rigid physiological algorithm. It is also notable that no patients in the Polish cohort were triaged as Green. This may reflect the specific dispatch criteria of the Polish EMS, or the nature of the calls encountered during the study period, focusing the comparison on more acute patients (Red/Yellow). Accordingly, κ = 1.00 should be interpreted in the context of this restricted category range (Red/Yellow only), which can inflate agreement metrics, and the fact that the observer was not blinded to the routine category.

#### Saudi Arabia: substantial agreement with CTAS (linear κ = 0.841; quadratic κ = 0.881)

The agreement between CTAS and the TTT is statistically strong, but the deviations are highly informative. The CTAS is a comprehensive five-level system that uses not only physiological parameters but also a wide array of first- and second-order modifiers based on the chief complaint (e.g., severe pain, mechanism of injury, high-risk situations)^17^. For the purposes of comparison with the three-level TTT output, CTAS categories were harmonized as CTAS 1 = Red, CTAS 2 = Yellow, and CTAS 3–5 = Green; this necessary collapse introduces assumptions that may influence observed agreement.

- **TTT overtriage (routine green → TTT yellow)**: Cases such as two children with fever, one Crohn’s diseases, one liver Cirrhosis, and one adult with headache, all indicate the existence of abnormal breathing or hyperventilation. This is a classic example of TTT’s physiological focus. While a CTAS-trained provider might use clinical judgment to classify a stable patient with fever and slightly fast breathing as Green (Level 4/5), the TTT’s algorithm appears to automatically escalate to Yellow based on the abnormal breathing, regardless of the cause^19,20^.
- **TTT undertriage (routine red → TTT yellow)**: Cases such as Testis torsion, Diabetic Ketoacidosis (DKA), Upper Gastrointestinal Bleeding (UGIB), etc. were all assigned a lower urgency category by the TTT in the absence of breathing difficulties. This is the most critical finding from a patient safety perspective. CTAS would classify these patients as Red (Level 1) or high Yellow (Level 2) based on the high-risk nature of the diagnosis (time-sensitive condition, potential for hemodynamic collapse), even if the patient’s breathing is currently normal. The TTT, by focusing on the *current* physiological state, misses the *anticipated* trajectory of these illnesses. It fails to account for the diagnostic urgency that is a core component of CTAS. However, in a disaster or public health emergency when resources and time are limited, these cases will probably be downgraded to rescue those in immediate need of assessment and interventions^7,19,20^.

#### Thailand: good agreement with ESI (κ = 0.701)

The agreement is still good, but lower than in Saudi Arabia, suggesting more frequent or significant deviations. The Emergency Severity Index (ESI) is also a five-level system that uniquely prioritizes two key questions before assessing vitals: (1) Does the patient require immediate life-saving intervention? (2) Is this a high-risk situation? For comparability with the three-level TTT output, ESI categories were harmonized as ESI 1 = Red, ESI 2 = Yellow, and ESI 3–5 = Green; as with CTAS, this collapse may influence observed agreement. TTT Undertriage (Most Significant Finding) was observed in some of the cases.

- **Red → Yellow**: Trauma, Syncope, Chest pain, Limb weakness (CVA). ESI would categorize these as high-risk presentations demanding immediate evaluation, making them at least a Level 2 (Red). The TTT downgrades them, likely because the patient’s vitals were transiently stable at the moment of assessment.^18,21^.
- **Red → Green**: Mental deterioration. A significant undertriage was noted. ESI would flag any patient with acute confusion or lethargy as high-risk (Level 2).
- **Yellow → Green**: Dialysis patient, Unconscious (but breathing). These are also concerning. An unconscious patient is almost always an ESI Level 1 or 2. A dialysis patient with any acute complaint is considered high-risk due to potential electrolyte or fluid balance issues.^18,21^.

In conclusion, while the TTT tended to assign lower urgency categories (relative to CTAS and ESI) for some patients who were physiologically stable “*at the moment*,” but had a high-risk chief complaint or underlying condition that would be flagged by the more sophisticated logic of CTAS and ESI, this pattern might be less important in a disaster and public health emergency where triage priorities and resource constraints differ from routine care. The use of five-level triage system may be impractical for high-volume sorting in many mass-casualty or resource-constrained settings and some of the discussed cases might be categorized differently under disaster triage principles that prioritize immediate, observable threats to life.

The context of a disaster or a public health emergency fundamentally changes the goals and constraints of triage^22^. Therefore, the interpretation of this study should be limited to post-hoc concordance in routine prehospital encounters, but the observed discordance patterns suggest that the perceived “weaknesses” of the TTT in routine settings could be consistent with the simplifying logic required in surge scenarios. However, these implications are hypothesis-generating only, because this study did not evaluate operational performance or patient outcomes in actual disasters or public health emergencies.

### Conceptual implications for surge and disaster settings (hypothesis-generating)

The fundamental shift in triage philosophy and approach depends highly on the severity of the event and the ethical framework of care. In a normal condition, the goal is to provide the best possible care for each individual patient. Triage systems like CTAS and ESI are designed to identify an individual’s risk and predict their resource needs to ensure they get timely care. In contrast, during disasters and public health emergencies, the goal shifts to doing the greatest good for the greatest number of people. This is a utilitarian public health approach^23^, when the system is overwhelmed, and resources (staff, beds, ventilators) are scarce. Triage is no longer about predicting an individual’s needs but about allocating profoundly limited resources to save the most lives possible^7,24^. Therefore, several other factors need to be considered in the evaluation of the tool. Importantly, the present study assessed post-hoc categorical concordance in routine prehospital encounters, and the implications below are conceptual and hypothesis-generating rather than outcome-based evidence.

#### Speed and simplicity become paramount

In a disaster, there is a massive influx of patients and a limited number of trained providers who are working under immense stress. The comprehensive, nuanced nature of CTAS and ESI becomes a liability.^17,18^ They are too slow to perform on dozens or hundreds of patients. The cognitive burden of remembering numerous modifiers and clinical subtleties is too high in a chaotic environment. On the other hand, the simplicity and speed of the TTT, mirroring the START methodology, become its most valuable asset. It can be applied rapidly by a wide range of personnel, including those who may be cross-trained or brought in as surge staff. The complete concordance observed with START-aligned triage in the Polish cohort is consistent with this interpretation; however, because the Polish sample included only Red/Yellow categories and observers were not blinded, this finding should be interpreted cautiously and viewed as supportive rather than definitive evidence of performance in a true MCI setting.^3,4,25^.

#### The TTT “undertriage” becomes “appropriate prioritization”

This is the most critical re-interpretation. In this original study, the TTT’s assignment of a lower urgency category (relative to CTAS/ESI) of a Red patient with DKA or Testis Torsion to Yellow was a significant safety concern. In a disaster, this is likely the *correct* allocation of resources. In a disaster, the Red tag is reserved for patients with an immediate threat to life who are also salvageable with a brief, life-saving intervention^22^. The trauma patient gets the Red tag. The DKA patient, while critically ill, is physiologically more stable *at that moment* and can better tolerate a delay than the trauma patient. The discordance patterns observed in this study suggest that the TTT algorithm may, in practice, make this difficult decision by prioritizing the patient’s immediate physiological state and sorting patients based on immediate physiological threats over diagnostic possibilities, which is the core principle of disaster triage^25–27^. However, because this study did not evaluate outcomes or operational performance in disasters, this interpretation remains hypothesis-generating.

#### The “overtriage” of the TTT becomes “effective screening” in a pandemic

During the COVID-19 pandemic, the primary challenge was identifying patients with respiratory compromise, especially those with “silent hypoxia” who appeared deceptively well. In Saudi Arabia, TTT “overtriaged” five Green patients to Yellow because of “abnormal breathing or hyperventilation.” In a respiratory pandemic, a tool that is highly sensitive to abnormalities in breathing may be advantagous. It would effectively flag patients at risk of deterioration from COVID-19, even if their initial complaint was simply “fever.” It prioritizes the cardinal symptom of the public health emergency, allowing for rapid isolation, and initiation of respiratory support (e.g., oxygen). CTAS or ESI might have classified these patients as Green, potentially sending them to a less-monitored area^25,26^. Nevertheless, this study was not conducted in a pandemic context and did not evaluate downstream outcomes; therefore, this implication should be interpreted as conceptual.

#### Resource availability

This analysis highlights the critical context of resource availability. High-income countries (HICs), such as two of the settings in our study, typically employ sophisticated, 5-level triage systems like CTAS and ESI. These systems are resource-predictive and depend on a robust infrastructure with highly trained providers and rapid diagnostic capabilities. Conversely, many low- and middle-income countries (LMICs) operate with significant resource constraints, including fewer trained personnel, limited equipment, and greater transport distances. In these settings, complex 5-level scales are often impractical and unsustainable for routine use. Simpler, more rapid, and purely physiologically based tools are often a necessity. While our study did not include a true low-income setting (Thailand is an upper-middle-income country), the TTT’s simple, physiological logic (which aligns with START) may, paradoxically, make it a *more* suitable candidate as a “translational” tool in LMIC settings than in HICs. Its failure to align with HIC *routine* triage (CTAS/ESI) may not be a weakness in a setting where START-like logic is already the standard of care due to resource necessity. This remains a critical area for future investigation^28,29^. In addition, because CTAS and ESI were harmonized from five levels to a three-level framework for comparison in this study, discordance with TTT should be interpreted in light of this necessary methodological simplification.

### The choice: TTT for disaster and pandemic response

Given the outcomes of this study, it is reasonable to consider the use of the TTT in a disaster or a resource-limited public health emergency, because:

1. It operates on the principle of immediate physiological state, which directly aligns with the disaster goal of identifying patients who will die without immediate, simple intervention.
2. It is fast, simple, and objective. It can be taught quickly and applied consistently under duress, reducing cognitive load on overwhelmed healthcare providers.
3. It appropriately filters patients, reserving the highest triage levels (Red) for those with the most immediate, fixable life-threats by focusing on the “now” (current breathing, circulation) rather than the “what if” (complex diagnostics, potential decline).
4. Its high sensitivity to respiratory abnormalities makes it an ideal screening tool during a pandemic like COVID-19, effectively identifying at-risk patients even at an early stage (Adaptability).

Therefore, this study, when viewed through the lens of a crisis, does not just show good agreement between tools. It suggests that the TTT’s physiologically driven algorithm is not a “dumbed-down” version of routine triage, but rather reflects a different triage philosophy that may align with the rapid sorting demands of disaster medicine^4,6,7^. However, because this study measured post-hoc concordance in routine care and did not evaluate disaster operations or patient outcomes, these implications should be considered hypothesis-generating and require dedicated prospective validation in surge or MCI settings.

In addition, it should be acknowledged that several new rapid major incident triage approaches have recently been proposed and, in some settings, recommended for use. One example is the Ten Second Triage (TST) tool, developed in the United Kingdom as part of an NHS England review of major incident triage, intended to enable very rapid categorization and early life-saving actions by responders at the scene. Conceptually, TST shares important similarities with physiologically oriented algorithms such as the TTT, including an emphasis on speed, simple observable criteria, and prioritization in the context of severely limited resources. However, while the TTT has been described through a staged research program (conceptual development, expert consensus, and simulation-based comparisons), the published evidence base for newer tools such as TST is still developing and includes descriptive publications and early evaluation work. Therefore, direct comparative studies and outcome-based validation remain important to clarify the relative performance, safety, and best-use contexts of these rapid triage systems^30–32^.

### Strengths of this study

The prospective and observational design of this study provides higher ecological validity than retrospective or simulation studies, because triage categorization was recorded during real prehospital encounters. Comparing three diverse healthcare systems and routine triage paradigms significantly enhances the generalizability and transferability of study’s findings, while also allowing the TTT to be examined against fundamentally different triage philosophies. Standardized training for TTT assessors and the independent, simultaneous application of the triage tools helped to standardize assessment and reduce variability in TTT application across sites. The use of Cohen’s Kappa (linear and quadratic) with 95% confidence intervals to quantify categorical agreement is appropriate for this type of ordinal triage data, and the inclusion of cross-tabulations enables transparent interpretation of the direction and clinical context of discordant assignments.

### Limitations and considerations

This study has several limitations. First and most critically, this was an observational concordance study, not an implementation trial. The TTT was applied post-hoc by an observer and was not used for clinical decision-making, precision, applicability or operational feasibility. Accordingly, this study does not validate the clinical accuracy or safety of the TTT, and it cannot determine outcome-based over- or under-triage.

Second, the observers were not blinded to the routine triage outcome. It is highly likely that knowledge of the EMS crew’s decision created a contamination bias, artificially inflating the agreement scores (Kappa). The true, unbiased concordance is likely lower than what we report. In addition, formal inter-rater reliability among multiple TTT assessors was not evaluated, and the reproducibility of the TTT assignment across different raters remains unknown.

Third, the data from the three sites are highly heterogeneous, representing different triage systems (START, CTAS, ESI), patient populations, and EMS models. While this was intentional to test the TTT against diverse systems, we have avoided a pooled statistical metric (like a total Kappa) as it is methodologically indefensible. Each site must be interpreted as a separate case study. Although an “overall” kappa was calculated on pooled data after harmonizing five-level scales into a three-level framework, this value is descriptive only and should be interpreted with caution because it aggregates heterogeneous tools and case-mix.

Finally, in Poland, the sample contained only Red/Yellow categories (no Green), which restricts variability and may inflate kappa estimates despite the observed complete concordance.

### Implications and recommendations for future research

1. **Defining the role of the TTT**: The results of this study suggest that the TTT is not a direct replacement for comprehensive systems like CTAS or ESI in a well-resourced hospital ED. Its strength lies in its simplicity and physiological focus, and thus, it can ideally be used:

- As a rapid primary triage approach to mass casualty incidents (MCI), similar philosophy to START.
- In low-resource settings where extensive provider training is not feasible.
- For use by first responders or staff with less clinical training to provide an initial, rapid assessment before a more detailed triage can occur.

However, these proposed roles remain hypothesis-generating, because the present study evaluated post-hoc concordance rather than operational performance or patient outcomes in disasters.

2. **A validation study**: The immediate priority should be a follow-up study that tracks patients from the field to a final hospital outcome, allowing to analyze the rates of under- and overtriage for the TTT *as defined by patient outcomes*, not just by comparison to another tool. This is the only way to establish its safety profile. Such a study should also allow assessment of subgroups (e.g., high-risk chief complaints with stable physiology) identified as discordant in this study.
3. **Investigate time and resources**: A future study should also measure the time it takes to perform triage with the TTT versus the routine systems. The TTT’s simplicity likely makes it faster, which is a significant advantage if it can be proven safe. Operational endpoints (time-to-triage, provider workload, training time) should be reported alongside outcome-based safety metrics.
4. **The use of AI**: The “diagnostic blind spot” in the TTT is a critical area for improvement, and artificial intelligence (AI) may hold significant promise in resolving it. Beyond triage, AI is already a rapidly expanding and crucial asset across all phases of disaster management, from predicting events and optimizing resource allocation to automating search and rescue efforts and improving communication, fundamentally bolstering our capacity to respond to MCIs and other emergencies. AI’s ability to analyze vast, complex datasets, identify subtle patterns, integrate real-time information, and continuously learn can enhance the TTT’s precision by reducing over- or under-triage for specific patient presentations, and would be an excellent idea for further exploration. AI-supported decision aids could potentially incorporate high-risk complaint modifiers while preserving the rapid, physiologically oriented structure needed for surge contexts.

## Conclusion

The TTT demonstrates site-dependent concordance with established routine triage systems. It aligns with complete concordance within the restricted Poland sample with the physiological, disaster-focused START tool, but shows clinically relevant deviations from complaint-/modifier-driven routine systems like CTAS and ESI. The TTT tended to assign lower urgency categories (relative to CTAS/ESI) for some high-risk patients (e.g., DKA, syncope, CVA) who were physiologically stable at the time of assessment. These discordances should be interpreted in light of the methodological need to harmonize CTAS and ESI from five levels into a three-level framework for comparison, and the fact that observers were not blinded.

Therefore, our findings indicate that the TTT, in its current form, is *not* a suitable “translational” tool for scalable use in *both* daily practice and MCIs. Its lack of sensitivity to high-risk chief complaints and diagnoses makes it unsafe as a replacement for routine primary triage. This study reinforces that the TTT’s logic is primarily that of a disaster-specific tool and highlights the fundamental challenge of creating a single, scalable system that can safely bridge the gap between routine and disaster triage. Future work should prioritize outcome-based validation and operational testing before any implementation recommendations are made.

## Supplementary Information

Below is the link to the electronic supplementary material.

Supplementary Material 1

Supplementary Material 2

## Data Availability

All data generated or analysed during this study are included in this published article, and its supplementary information files.
