# Supplementary material for: A multi-national observational study on the concordance between the translational triage tool and routine prehospital triage
Source: Sci Rep. 2026 May 7;16:14645. doi: 10.1038/s41598-026-52015-7 (PMC13153196; doi:10.1038/s41598-026-52015-7)
Supplement: Supplementary file 1 — Supplementary Material 1 [file 41598_2026_52015_MOESM1_ESM.pdf]

CHULABHORN
ROYAL ACADEMY

แบบเอกสารที่ ECF-CRA 20

Human Research Ethics Committee
Chulabhorn Royal Academy

Title of Project	A comparative study of translational triage tools in emergency situations and standard triage tools in normal situations
Project Code	EC 139/2567
Principal Investigator	Faculty of Health Science Technology
Affiliation	HRH Princess Chulabhorn College of Medical Science
Date of Approval	January 17, 2025
Approval Period	1 year

The aforementioned documents have been reviewed and approved by Human Research Ethics Committee, based on the Declaration of Helsinki.

Signature Chaivej Nuchprayoon

(Professor Emeritus Chaivej Nuchprayoon, M.D.)
Chairperson
Human Research Ethics Committee
