# Supplementary material for: A multi-national observational study on the concordance between the translational triage tool and routine prehospital triage
Source: Sci Rep. 2026 May 7;16:14645. doi: 10.1038/s41598-026-52015-7 (PMC13153196; doi:10.1038/s41598-026-52015-7)
Supplement: Supplementary file 2 — Supplementary Material 2 [file 41598_2026_52015_MOESM2_ESM.pdf]

تجمع جازان الصحي
لجنة أخلاقيات البحوث

الموافقة تشمل مركز الأزمات والكوارث بتجمع جازان الصحي.
ونود التنويه بأن دعوة الممارسين الصحيين والموظفين من منسوبي المنشأة للمشاركة في البحث يكون عن طريق مقابلتهم في المنشأة أو تعميم من الإدارة لديكم بأي وسيلة ترونها مناسبة ان أمكن ، حيث يمنع إعطاء الباحثين أرقام التواصل والإيميلات الشخصية الخاصة بالموظفين إلا بعد موافقتهم الخطية للباحث على ذلك.

Approval Letter

**Date**	24/12/2024
**Principal Investigator**	Dr. Zakaria Mani.
**Protocol Title**	The use of Translational Triage Tools in emergencies- A quantitative, comparative study in pre-hospital settings (TTT # IV).
**Approval Number**	No. 24113
**Study Settings**	MOH Pre-hospital Care.

Dear Dr. Zakaria Mani:

The Jazan Ethics Committee is pleased to inform you that the above-mentioned study has been reviewed and approved.

<u>Approval Conditions</u>

- This approval is valid for one year, starting from the date of this letter.
- Should any amendments need to be made to the study, the principal investigator is required to request prior approval from the Jazan Ethics Committee.
- This letter provides you with ethical clearance to implement your study; however, you still need to obtain administrative approval from the site/s where the study will be conducted.
- If the research will not be completed in time, the principal investigator can request an extension letter from the Jazan Ethics Committee one month before the expiry of the approval.
- Documents related to the study should be kept by the principal investigator for five years after the completion of the study.
- The principal investigator is required to submit a progress report to the Jazan Ethics Committee every six months.
- At the end of the study, principal investigator is required to submit a final study report, including the manuscript intended for publication, both to the Jazan Ethics Committee and the database at www.marifah.gov.sa.

The Jazan Ethics Committee thanks you for submitting your study for review and wishes you all the best.

Sincerely,
Mrs. Fatma Rajhi

Chairman of Jazan Ethics Committee,
Jazan Health Cluster , Saudi Arabia

تجمع جازان الصحي
JAZAN HEALTH CLUSTER
لجنة أخلاقيات البحوث
Research Ethics Committee
